# Enhanced Transformation of TNT by Arabidopsis Plants Expressing an Old Yellow Enzyme

**DOI:** 10.1371/journal.pone.0039861

**Published:** 2012-07-11

**Authors:** Bo Zhu, Ri-He Peng, Xiao-Yan Fu, Xiao-Fen Jin, Wei Zhao, Jing Xu, Hong-Juan Han, Jian-Jie Gao, Zhi-Sheng Xu, Lin Bian, Quan-Hong Yao

**Affiliations:** 1 Agro-Biotechnology Research Center, Shanghai Academy of Agricultural Sciences, Shanghai, China; 2 College of Horticulture, Nanjing Agricultural University, Nanjing, China; 3 College of Bioscience and Biotechnology, Yangzhou University, Yangzhou, China; United States Department of Agriculture, Agricultural Research Service, United States of America

## Abstract

2,4,6-Trinitrotoluene (TNT) is released in nature from manufacturing or demilitarization facilities, as well as after the firing or detonation of munitions or leakage from explosive remnants of war. Environmental contamination by TNT is associated with human health risks, necessitating the development of cost-effective remediation techniques. The lack of affordable and effective cleanup technologies for explosives contamination requires the development of better processes. In this study, we present a system for TNT phytoremediation by overexpressing the old yellow enzyme (*OYE3)* gene from *Saccharomyces cerevisiae*. The resulting transgenic Arabidopsis plants demonstrated significantly enhanced TNT tolerances and a strikingly higher capacity to remove TNT from their media. The current work indicates that *S. cerevisiae OYE3* overexpression in Arabidopsis is an efficient method for the phytoremoval and degradation of TNT. Our findings have the potential to provide a suitable remediation strategy for sites contaminated by TNT.

## Introduction

2,4,6-Trinitrotoluene (TNT) and other nitroaromatics have been detected at varying concentrations in munitions production facilities and military training and testing sites worldwide [1]–[4]. The areas of former TNT production plants are often contaminated with this explosive and its precursors, which are toxic [5] and mutagenic [6].

Phytoremediation, the use of plants to remove environmental pollutants, offers a low-cost, sustainable alternative to conventional remediation technologies and is attracting considerable attention as a means of cleaning up sites contaminated with explosives [7]. TNT is a primary concern for remediation because of its toxicity to humans (it is designateda as a class C carcinogen) and the extent of environmental contamination [8]. Phytoremediation has several forms. Phytoextraction removes metals or organic materials from soils by accumulating them in the biomass of plants. Phytodegradation or phytotransformation is the use of plants to uptake, store, and degrade organic pollutants. Rhizofiltration involves the removal of pollutants from aqueous sources through absorption via plant roots. Phytostabilization reduces the bioavailability of pollutants by immobilizing or binding them to the soil matrix, and phytovolatilization uses plants to take pollutants from the growth matrix, transform them, and release them into the atmosphere [9].

Genetic engineering has efficiently improved phytoremediation through a transgenic approach. The phytoremediation of explosives such as TNT, nitroglycerin, pentaerythritol tetranitrate and various heavy metals using plant cell cultures and transgenic plants is the current field of biotechnology that is receiving much attention by researchers and is gaining public acceptance. Phytoremediation can be a highly effective solar–powered, “green solution” for reducing risk to human and ecosystem health from pesticide-contaminated soil [10].

Old yellow enzyme (OYE) was first isolated from brewers’ bottom yeast by Warburg and Christian (1932) [11], was first purified by Theorell in 1935 and consists of a colourless apoprotein and a yellow dye, both essential for enzyme activity [12]. The discovery of OYE components helped to establish the essential role of protein in enzyme catalysis. OYE is similar to vitamin B_2_ (riboflavin). Thus, OYE provided the first biochemical role for a vitamin. In Williams’ paper, they have reported that Theorell demonstrated that the yellow cofactor was in fact riboflavin 5′- phosphate, now called flavin mononucleotide (FMN) [13]. In 1995, OYE3 was cloned from S*accharomyces* cerevisiae by Niino et al. and they also analyzed the OYE3 protein expressed in Escherichia coli. OYE2 and OYE3 are closely related, and they show differences in their ligand-binding properties and in their catalytic activities with oxygen and cyclohexen-2-one as acceptors [14].

The purpose of this study is to introduce OYE3 of the *S. cerevisiae* system into plants and determine whether the transgenic plants are able to degrade TNT more effectively than wild-type plants. We found that the transformed plants showed significantly elevated tolerance to TNT stress. Our physiological studies show unambiguously that the ectopic expression of *OYE3* gene in Arabidopsis results in enhanced TNT tolerance.

## Results

### Synthesis of *OYE3* from *S. cerevisiae*

We synthesized *OYE3* based on the encoding amino acid of the wild-type gene from *S. cerevisiae* (GenBank Accession No: BK006949). Twenty-nine 60 nt oligonucleotides and one 61 nt oligonucleotide were used to synthesize the recombinant gene (Table S1). The experimental details of the overlap extension polymerase chain reaction (OE-PCR) are outlined in Fig. S1. A BLAST search showed that the synthesized recombinant *OYE3* was 100% identical to the wild- type *OYE3* gene.

### Construction of Transgenic Arabidopsis

The synthesized *OYE3* was inserted into the binary vector pCAMBIA-1301 under the control of the cauliflower mosaic virus 35S promoter, and the construct was verified by sequencing, then introduced into Arabidopsis via Agrobacterium (GV3101)-mediated transformation. Ten transgenic plants (T1) were initially identified by PCR from 16 putative transgenic plants regenerated on half-strength MS [15] agar plates containing hygromycin. All of the transgenic plants were identical to the wild-type plants in phenotype when grown either on half-strength MS agar plates or in soil in a growth room, which suggests that the insertion of the *OYE3* in these plants caused no visible morphologic changes.

### Stable *OYE3* Expression in Transgenic Arabidopsis

**Figure 1:**
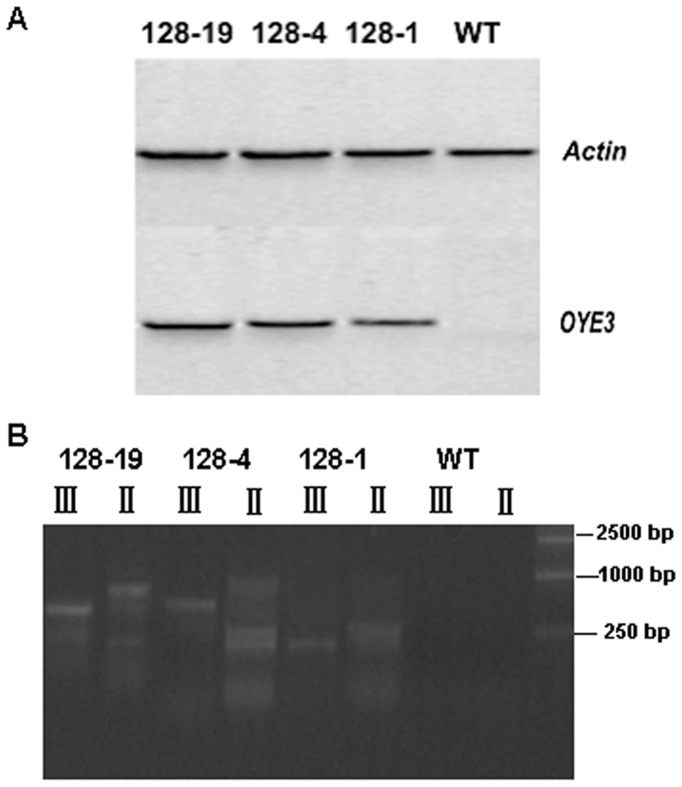
PCR analysis of the OYE3 gene fragment. A, RT-PCR analysis of the OYE3 gene fragment, WT serves as wild-type plant; wild-type and AH128 lines (AH128-1,-4,-19) with actin gene as a reference.B, Agarose gel analysis of TAIL-PCR products amplified from transgenic lines and wild-type plants. AD1 was used for lines AH128-1, AH128-4 and AH128-19. Lane designations II and III indicate products of the secondary and complete tertiary reactions respectively and WT serves as wild-type plant.

The homozygous transgenic lines expressing *OYE3* were selected from the T3 plants using hygromycin selection and reverse transcription polymerase chain reaction (RT-PCR). An RT-PCR product of approximately 220 bp in the 3′ domain of the OYE3 coding sequence was detected in three lines analyzed, whereas no such signals were detected in wild-type plants (Fig. 1A). Moreover, Tail-PCR method was used for detecting whether the transgenic lines were inserted independently. From Fig. 1B, we could observe there were detected bands in secondary and tertiary PCR for three lines, but no band was observed in wild-type plants. The lengths of the LB (left border) flanking sequences of the T-DNA were 240 bp for AH128-1, 746 bp for AH128-4 and 720 bp for AH128-19. It was found that three flanking sequences were inserted at different point by blast and maps analysis. No any coding region sequence was found for flanking sequences’ blast in GenBank database. This showed that the T-DNA was not inserted in the coding region of the known T-DNA insertion and did not cause damage on some of the known genes. It was found that three transgenic lines belonged to different lines by blast among these flanking sequences. These results could prove that the three transgenic lines were independent insertion.

### Enhanced Tolerance of Transgenic Plants to TNT on Plates and Flasks

**Figure 2:**
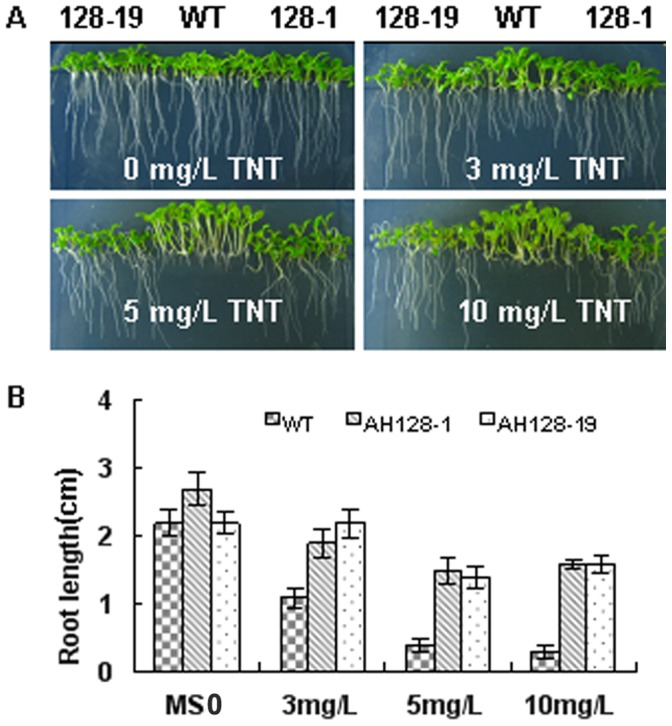
Enhanced TNT tolerances of plants on plates. A, WT and AH128 plants germinated and grown vertically for 2 weeks on half-strength MS agar plates containing 0.0, 3, 5, or 10 mg/L TNT. B, Root length of 2-week-old WT and AH128 plants grown on half-strength MS agar plates containing 0.0, 3, 5, or 10 mg/L TNT. The data represent mean ±SD (n = 10).

Seeds from each wild-type and transgenic lines were sown on half-strength MS agar plates containing 0, 3, 5 and 10 mg/L TNT, and grown vertically for 2 weeks. Both wild-type and transgenic plants developed similar root systems with root hairs on the control medium without TNT. On the medium containing 3 mg/L TNT for 2 weeks, transgenic plants displayed minimal sign of phytotoxic effects, showing a normal root development, while wild-type seedlings exhibited a shorter root development. At 5 mg/L TNT, transgenic plants could still develop gradually to later stages and produced longer roots and stronger green leaves than wild-type plants. At 10 mg/L TNT, the average root length of transgenic plants was 1.6 cm. However, the average root length of wild-type plants was about 20%of those of transgenic plants (Fig. 2A, B). Efficient root and shoot formation is a crucial characteristic of transgenic plants designed for potential application in phytoremediation. In figure 2A, we could observe that the wild-type plants had stronger shoot formation than transgenic plants in 3, 5, 10 mg/L TNT media, especially in 5 mg/L concentration; but the shoots were more friable and the leaves were less greener in the wild-type plants than in transgenic plants.

**Figure 3:**
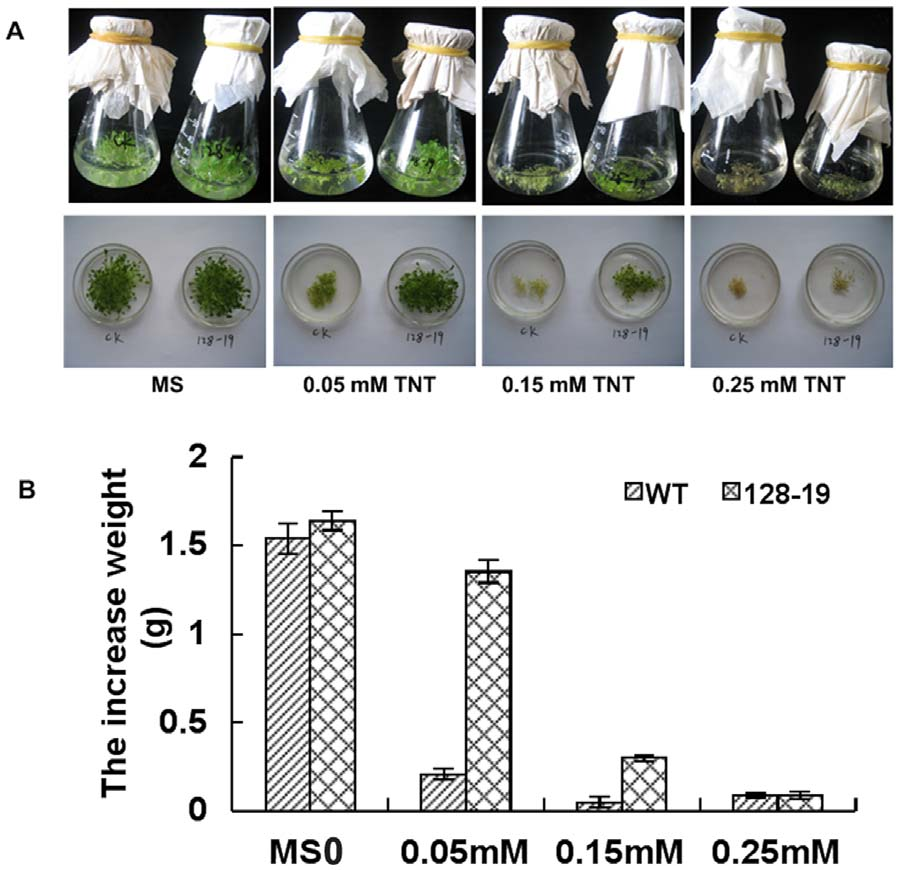
Growth of wild-type and transgenic line in liquid medium. A, Fifty seedlings grown in MS solid medium for 2 weeks were transferred to MS liquid medium in flasks containing 0, 0.05, 0.15 and 0.25 mM TNT. B, Increased fresh weights of seedlings in the individual flasks in the above treatment. The data represent mean ±SD (n = 3).

A toxicity study was conducted to determine the effect of TNT on wild-type and transgenic seedlings. The wild-type and AH128-19 seedlings grown without TNT in the liquid medium appeared healthy, which indicated that the toxic effects observed were only due to the presence of TNT and not the result of submersion in liquid medium. The increased wet weight rates for the wild-type and AH128-19 line plants were nearly the same, 92.49% for wild-type plants, and 92.50% for the latter. At 0.05 mM TNT, however, the growth of the wild-type plants was significantly inhibited, with the average fresh weight being about 0.2 g per flask, about 16% of those of AH128-19. At 0.15 mM, only some of the wild-type plantlets could survive the incubation, producing a final fresh weight of 0.05 g. In contrast, the seedlings of the transgenic plants showed a relatively normal morphology and growth rate, producing an average final fresh weight of approximately 0.3 g. A toxicity study was also conducted at 0.25 mM TNT. The wild-type seedlings almost died and the AH128-19 plants were likewise seriously damaged, but at a lesser degree than the wild-type plants (Fig. 3A, B).

**Figure 4:**
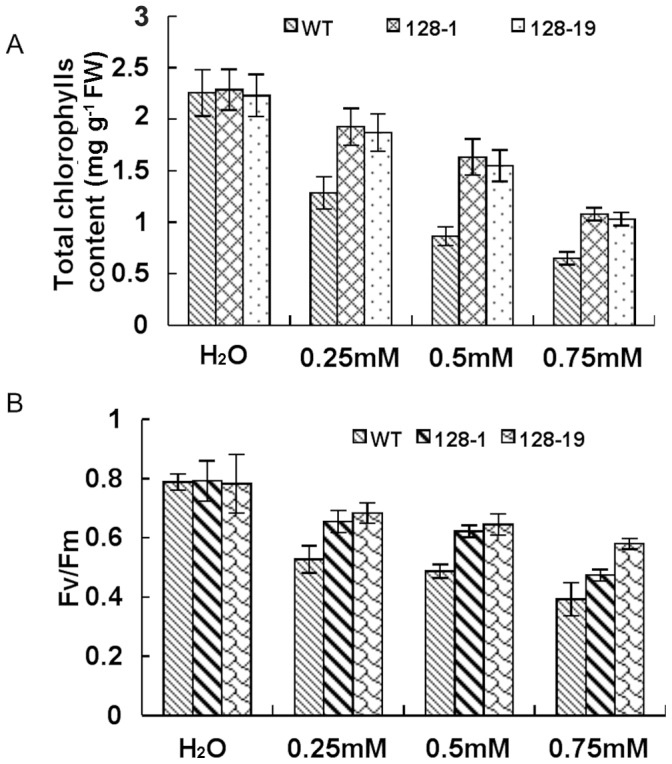
Effect of TNT treatment on photosynthetic activities. A, Chlorophyll (Chl) changes in wild-type and transformed plants. B, Fv/Fm changes in wild-type and transformed plants. Wild-type and transformed plants grown in soil for 30 days and then soaked with 0, 0.25, 0.5 and 0.75 mM TNT for a week. The data represent mean ±SD (n  = 3).

We selected two transgenic lines (128-1 and 128-19) and the wild-type plants for the subsequent experiments. The total chlorophyll content in the wild-type and transgenic plants were markedly decreased after stress. However, the decrease in the transgenic plants was significantly lower than that in the wild-type plants (Fig. 4A). Chlorophyll fluorescence has been routinely used for many years to monitor the photosynthetic performance of plants non-invasively and to screen plants for tolerance to environmental stresses [16]. TNT damage on the *OYE3*-expressing plants was evaluated both as photosystem II (PSII) stability and as injury to the whole plant. Fv/Fm was used to estimate the quantum yield of PSII photochemistry. The wild-type and transgenic plants were exposed to excess of light energy at high temperature, and their Fv/Fm was measured at different TNT concentrations. High TNT concentrations caused marked inhibition of PSII, as indicated by decreases in the Fv/Fm value. However, the Fv/Fm value for the wild-type plants was dramatically lower than those of transgenic plants after TNT stress (Fig. 4B). This is consistent with the hypothesis that plants overexpressing *OYE3* are more tolerant of photoinhibition than wild-type plants.

### High-performance Liquid Chromatography (HPLC) Analysis of TNT Degradation

**Figure 5:**
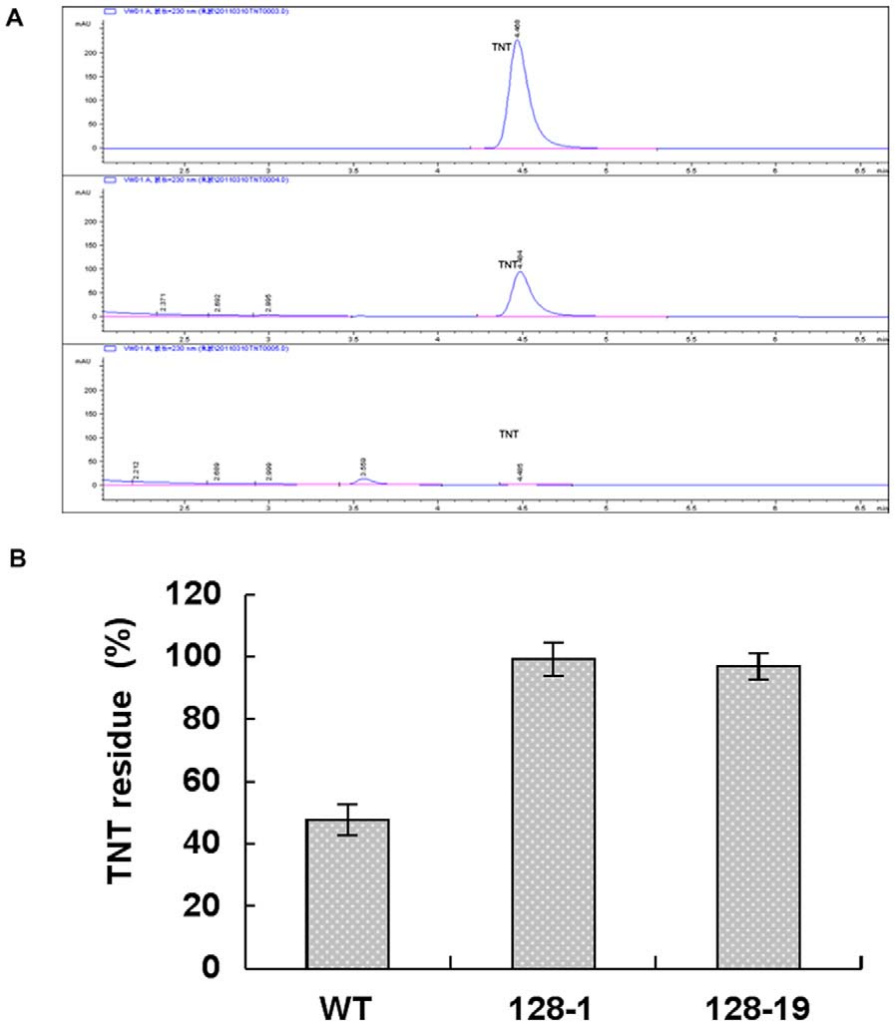
Analysis of OYE3 enzyme properties. A, HPLC analyses of residual TNT in seedlings. B, TNT degradation rates in vivo for wild-type and transgenic plants.

We extracted crude protein from wild-type and transgenic seedlings and performed a vivo assay by using high-performance liquid chromatography (HPLC). HPLC analysis was used to detect whether the TNT was actually degraded. The chromatogram of the control sample that contained only TNT showed a single peak at a retention time of 4.5 min. In the wild-type sample that contained TNT and wild-type extracts, a TNT peak was observed at a retention time of 4.5 min. The chromatogram of the transgenic line sample that contained TNT and transgenic line extracts showed a peak at the retention time of 3.5 min and no peak at the retention of 4.5 min. HPLC analysis revealed that detectable novel compounds accumulated in the extracts of the OYE3 plants (Fig. 5A). Indeed, OYE3 plants generally showed more than 2 times higher degradation rates than wild-type plants. The low levels of TNT found in the transgenic plant line and the increased resistance to toxicity strongly suggested that OYE3 degrades TNT *in planta*. (Fig. 5B).

## Discussion

Phytoremediation is an eco friendly approach for remediation of contaminated soil and water using plants [17]. Phytoremediation for environmental cleanup has been long recognized as a solution and it is defined as the use of plants for removal of environmental pollutants or detoxification to make them harmless [18], [19]. Enhancing plant tolerance to abiotic stresses involves multiple mechanisms and different physiological and biochemical pathways [20], [21]. Different strategies have been implemented to improve tolerance in crop plants, and transgenic plants are a powerful and promising approach [22].

In the present study, we have shown that plants grown under TNT-polluted substrates or atmosphere are able to take up these compounds through roots and cuticle, and bioaccumulate them in their tissues. However, the wild-type plants grown in TNT-contaminated media exhibited generally shorter roots, and even symptoms of cell death. The proposed approach showed that expression of the OYE3 gene from *S. cerevisiae* increases the tolerance of plants to TNT by conferring the ability to degrade these compounds.

The seeds from transgenic plants germinated and grew in media containing 3, 5 and 10 mg/L TNT (Fig. 2A, B), whereas the root growth of the wild-type seeds was severely inhibited under these conditions. Explosive residues are therefore potentially toxic to plant life and the expression of OYE explosive-degrading enzyme overcomes this toxicity. The TNT levels tolerated by the seedlings were significantly higher than those tolerated by germinating seeds, which implies that the inhibitory effect of TNT on the wild-type Arabidopsis seedlings is more significant at germination or the increased biomass and possibly elevated enzyme expression in older seedlings allowed for more effective detoxification of TNT. Many scholars have reported that TNT and its metabolites have an inhibitory effect on plant growth, resulting in the stunting of both root and shoot development [23], [24]. The current results are similar to those previously reported on this point. The weight studies (Fig. 3A) revealed that the transgenic plants had reduced growth rates in the presence of TNT. However, the normal formation of the seedlings in the presence of TNT (Fig. 3B) indicates that the transgenic plants are much more tolerant to TNT than the wild-type line.

The phytotoxic effects were evident in the extreme bleaching of the seedlings (Fig. 2A, B) and the termination of TNT transformation (Fig. 3A, B). In contrast, Arabidopsis seedlings that expressed *OYE3* were very efficient in removing all the TNT during the experiment. No TNT was extracted from the transgenic seedlings, which indicated that it was either completely transformed or sequestered within the plant in inextricable form (Fig. 4). Plants are known to conjugate xenobiotic compounds with sugars, glutathione, amino acids, and malonic acid as part of their detoxification strategy [25]. The conjugates are then compartmentalized in the vacuole as cell wall material or lignin. Reports have suggested that this also occurs with TNT [26], [27] and its metabolites, explaining the difficulty most researchers have in extracting TNT and its metabolites from plant material [23], [28]. The uptake and conjugation of TNT and its metabolites by the transgenic seedlings has great potential for phytoremediation of TNT from the environment and binding it in the plant material before composting or harvesting and removal.

In conclusion, transgenic plants expressing OYE3 have an enhanced ability to tolerate TNT phytotoxicity and detoxify its effects. Further investigations are underway to determine the ability of these transgenic plants to remove TNT from soil. The enhanced metabolism in transgenic Arabidopsis indicates that the introduction of OYE3 into fast-growing deep-rooted trees such as poplars, which are more suitable for phytoremediation purposes, could significantly increase TNT removal in the field. Such technology may provide affordable and effective remediation systems that are urgently required.

## Materials and Methods

### Design and Chemical Synthesis of the *OYE3* Gene

We have biosynthesized the recombinant gene OYE3 from *Saccharomyces cerevisiae*. PCR reactions were carried out with 1.5 pmol of each inner oligonucleotides and 30 pmol of outer oligonucleotides for 25 cycles with 2.5 U *pyrobest* polymerase (Takara, Dalian, China). The overlap extension PCR method was used to synthesize *OYE3* [29]. The conditions for the PCR-mediated assembly was 30 s at 94°C, 30 s at 45°C, and 30 s at 72°C for each cycle, followed by an additional 10 min at 72°C to ensure complete extension for all PCR reactions.

### Vector Construction and Plant Transformation

The A. thaliana cv. Columbia was transformed using Agrobacterium tumefaciens strain GV3101 using the floral dip method as described previously [30]. The plasmid used in the transformation was derived from pYF7716 [31]. The synthesized *OYE3* cDNA was digested with *Bam* HI and *Sac* I, and cloned into the binary vector under the control of an enhanced double CaMV 35S promoter and the tobacco mosaic virus TMV Omega leader sequence.

### Plant Materials and Growth Conditions

Seeds of *A. thaliana* were surface-sterilized with bleaching powder (5%, w/v) for 20 min, washed with sterile water three times, and placed in Petri dishes containing MS medium with 0.8% agar. The plates were incubated at 4°C for 2 days in darkness for the synchronization of germination. The seeds were incubated at 22°C in growth chambers under constant illumination with cool-white fluorescent lamps (70 µmol photon m^−2^ s^−1^) for 16 h and a dark period of 8 h daily. *A. thaliana* was grown on germination medium pots for about 2 weeks.

### PCR Analysis

Total RNA was digested with DNase I (Promega, Madison, WI, USA) to remove genomic DNA. The first strand of cDNA was synthesized by 5 µg of total RNA as template with the Reverse Transcription System (Promega, Madison, WI, USA) in a 20 µl reaction volume. To improve the reliability of the RT-PCR, the *A. thaliana actin* gene (AtAc2, accession number NM112764) was synthesized by two primers (AtAc2Z1∶5′-GCA CCC TGT TCT TCT TAC CGA G-3′; AtAc2F1∶5′-AGT AAG GTC ACG TCC AGC AAG G-3′) as the internal standard gene. The PCR was carried out as follows: 27 cycles of 40 s at 94°C, 30 s at 58°C, and 20 s at 72°C, plus a final extension at 72°C for 5 min. A 220 bp fragment of the OYE3 gene was amplified by two specific primers (OYE3Z1∶5′-GGA TCC ATG CCA TTT GTA AAA GGT TTT GAG CCG ATC-3′; OYE3 F1∶5′-GAG CTC AGT TCT TGT TCC AAC CTA AAT CTA C-3′) according to the O*YE3* sequence. The PCR conditions were the same as AtAc2. The PCR products were separated on 2% agarose gel and quantified by a Model Gel Doc 1000 (Bio-Rad, USA). DNA intensity ratio of *OYE3* to AtAc2 was analyzed with ShineTech Gel Analyzer (Shanghai Shine Science of Technology Co., Ltd, China) to evaluate the expression pattern of *OYE3*. The experiments were repeated three times with the same results, and one of them is presented.

Genomic DNA samples from Arabidopsis used for Tail-PCR analysis were extracted and purified from 1 g of fresh leaves according to the cetyltrimethylammonium bromide (CTAB) protocol [32] as modified by Luo et al. [33]. DNA quality was checked spectrophotometrically with a Biophotometer (Eppendorf) and analyzed by means of 0.8% agarose gel electrophoresis in 1× Tris-borate-EDTA stained with ethidium bromide. Specific primer sets complementary to the sequences flanking the cloning site left border region of transfer vector were: SP1∶5′-ATT CCT AAA ACC AAA ATC CAG TAC-3′; SP2∶5′-TTC TCC ATA ATA ATG TGT GAG TAG-3′; SP3∶5′-TCT GGA CCG ATG GCT GTG TAG AAG-3′. The three arbitrary degenera (AD) primers were used in combination with the specific primers for TAIL-PCR: AD1∶5′-NTC GAS TWT SGW GTT-3′; AD2∶5′-NGT CGA SWG ANA WGA A-3′; AD3∶5′-WGT GNA GWA NCA NAG A-3′. The specific primers were designed to have 60°C Tm higher than those of AD primers. PCR procedures were the same as described by Liu et al. [34]. The PCR products were purified and cloned into the PMD18-T vector (Takata, China) and then sequenced. The sequences were confirmed by BLAST in NCBI database.

### Toxicity Experiments

The transgenic and wild-type seeds were surface-sterilized, germinated on Murashige & Skoog (MS) complete medium plates amended with 0, 3, 5 and 10 mg/L TNT, and grown vertically for 2 weeks. Meanwhile, other wild-type and transgenic seeds were surface-sterilized and germinated in MS complete medium and grown for 10 days. The seedlings were rinsed in ultrahigh-pressure autoclaved water, weighed, and equal amounts of the biomass were aseptically transferred to sterile flasks containing 100 ml water. The seedlings were washed and transferred aseptically into 50 ml conical flasks containing 0.05 mM, 0.15 mM, and 0.25 mM TNT in sterile MS liquid medium. The flasks were incubated under light at 23°C with rotary shaking. The wet weight of the seedlings was then determined after a further 14 d of growth.

### Plant Resistance Assay

The 30-day-old plants grown in soil were soaked in another black plastic cups with 0, 0.25, 0.5 and 0.75 mM TNT for a week to assay the effect of TNT on photosynthesis. The plants could absorb the TNT through the roots and the leaves grew normally. Three to five Columbia ecotype leaves were used for photosynthesis analysis. Chlorophyll was extracted from individual leaves with 95% ethanol. The chlorophyll content was determined spectrophotometrically at 470, 649, and 665 nm according to Lichtenthaler [35]. The photosynthetic parameters were detected by an LI-6200 portable photosynthesis system (Li-Cor Inc., Lincoln, NE) and calculated as previously described [16]. The Fv and Fm parameters were determined after 30 min in the dark, and the light-adapted values (Fv′ and Fm′) were measured after 30 min of illumination with 500 µmol·m^−2^·s^−1^.

### Enzyme Extraction and Measurement of TNT by HPLC

For the extraction of *A. thaliana* crude protein, fresh leaf tissue (0.05 g) was homogenized in 0.5 ml of chilled 50 mM Tris-HCl (pH 7.4) containing 100 mM KAC, 1 mM dithiothreitol, 1 mM EDTA, and 20% vol/vol glycerol. The homogenate was filtered with two layers of muslin cloth and centrifuged at 13,000×g for 30 min in a refrigerated centrifuge at 4°C. Crude enzyme was quantified using Coomassie Brilliant Blue G250 and used for the in vitro activity assay. The reaction mixtures contained 100 µl of extracted crude protein from the leaves of the transgenic and wild-type plants, 0.02 mM NADH, 0.02 mM FMN, and 0.02 mM TNT dissolved in acetone. After incubation at 30°C for 60 min, the reaction was stopped with the addition of 20 µl of trichloroacetic acid (240 mg/mL). The resulting mixture was quick-frozen and stored at −70°C before HPLC analysis. Reverse-phase HPLC was performed with an Agilent 1100 HPLC system (Agilent Technologies, CA, USA) and a Columbus 5 µm C18 column (250 mm×4.60 mm, Phenomenex). Acetonitrile and H2O in 60%: 40% (v/v) proportion, were used as the eluant at a flow rate of 1.0 ml/min. The effluent from the column was monitored simultaneously at 230 nm by a diode array detector. A 20 µl aliquot of each sample was injected onto the column and the retention times of known standards were applied to identify TNT.

## Supporting Information

Figure S1 **The strategy for the chemical synthesis of the** ***OYE3*** **gene.** Red arrowhead denotes forward primer; Blue arrowhead denotes reverse primer.
(TIF)

Table S1 **Primers for enzymatic synthesis of the** ***OYE3*** **gene.** Odd number: forward primer, Even number: reverse primer, Boldface: *Bam* HI and *Sac* I sites.
(DOC)
