# Supplementary figures and images for: Enhanced Transformation of TNT by Arabidopsis Plants Expressing an Old Yellow Enzyme
Source: PLoS One. 2012 Jul 11;7(7):e39861. doi: 10.1371/journal.pone.0039861 (PMC3394746; doi:10.1371/journal.pone.0039861)

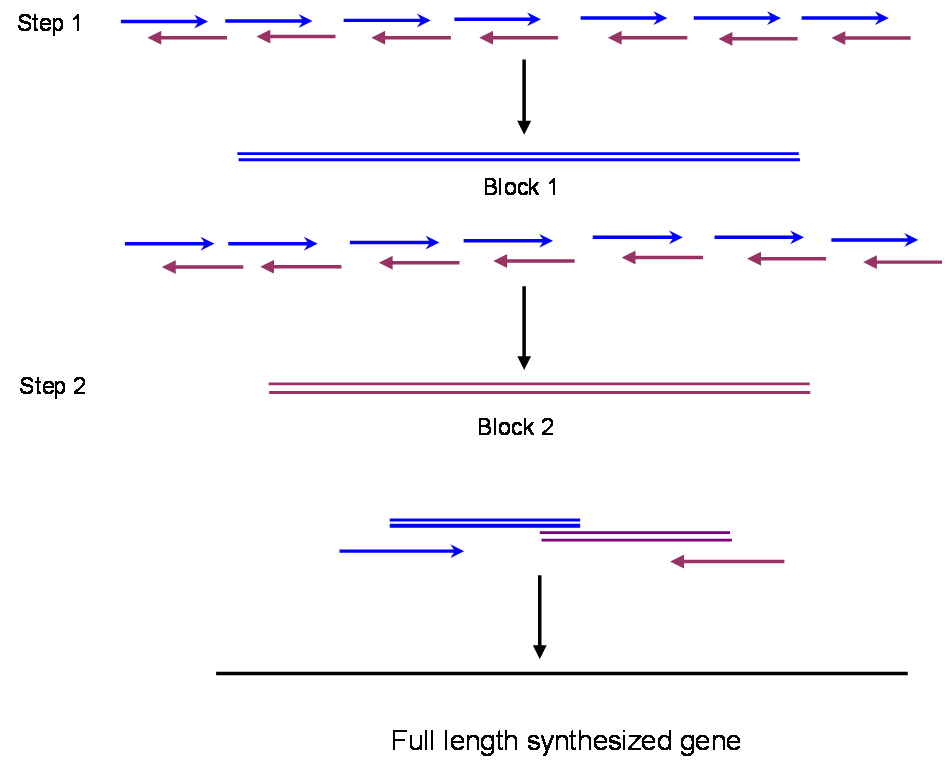

Supplement: Figure S1 — The strategy for the chemical synthesis of the OYE3 gene. Red arrowhead denotes forward primer; Blue arrowhead denotes reverse primer. (TIF) [file pone.0039861.s001.tif]
