# Supplementary material for: Enhanced Transformation of TNT by Arabidopsis Plants Expressing an Old Yellow Enzyme
Source: PLoS One. 2012 Jul 11;7(7):e39861. doi: 10.1371/journal.pone.0039861 (PMC3394746; doi:10.1371/journal.pone.0039861)
Supplement: Table S1 — Primers for enzymatic synthesis of the OYE3 gene. Odd number: forward primer, Even number: reverse primer, Boldface: Bam HI and Sac I sites. (DOC) [file pone.0039861.s002.doc]

**Table S1** **Primers for enzymatic synthesis of the *OYE3* gene**.

| Primer | Oligonucleotides |
| --- | --- |
| *OYE3*-1 | GAA**,GGA**,**TCC**,ATG,CCA,TTT,GTA,AAA,GGT,TTT,GAG,CCG,ATC,TCC,CTA,AGA,GAC,ACA,AAC,CTT |
| *OYE3*-2 | GAT,GTG,CAA,GCT,GAG,TGT,TAC,CAA,TCT,TAA,TTG,GTT,CAA,AAA,GGT,TTG,TGT,CTC,TTA,GGG |
| *OYE3-*3 | TAA,CAC,TCA,GCT,TGC,ACA,TCG,TGC,GGT,TAT,GCC,CCC,ATT,GAC,CAG,AAT,GAG,GGC,CAC,TCA |
| *OYE3*-4 | ATA,CAC,AGC,AGC,CCA,CTC,CTT,ATT,TGG,AAT,ATT,TCC,GGG,GTG,AGT,GGC,CCT,CAT,TCT,GGT |
| *OYE3*-5 | AGG,AGT,GGG,CTG,CTG,TGT,ATT,ATG,GTC,AGC,GTG,CTC,AAA,GAC,CTG,GTA,CCA,TGA,TCA,TCA |
| *OYE3*-6 | TCA,TAG,CCG,CCG,GCT,TGA,GGG,GAA,ATA,AAC,GTA,CCT,TCC,GTG,ATG,ATC,ATG,GTA,CCA,GGT |
| *OYE3*-7 | CCT,CAA,GCC,GGC,GGC,TAT,GAC,AAC,GCC,CCT,GGG,ATT,TGG,TCT,GAT,GAG,CAG,GTC,GCT,GAG |
| *OYE3*-8 | ACG,ACT,GAC,AAT,CAT,GGA,TGG,CTA,AAA,AGA,TAT,TCT,TCC,ACT,CAG,CGA,CCT,GCT,CAT,CAG |
| *OYE3*-9 | CAT,CCA,TGA,TTG,TCA,GTC,GTT,CGC,GTG,GGT,ACA,ACT,TTG,GTC,TTT,AGG,CTG,GGC,ATC,CTT |
| *OYE3*-10 | ACA,GTC,ATA,GCG,TAA,CCC,GTC,TCT,TGC,CAA,TAC,GTC,TGG,GAA,GGA,TGC,CCA,GCC,TAA,AGA |
| *OYE3*-11 | ACG,GGT,TAC,GCT,ATG,ACT,GTG,CAT,CTG,ACA,GAG,TGT,ATA,TGA,ATG,CTA,CGT,TAC,AAG,AAA |
| *OYE3*-12 | TTA,GTC,AAA,CTA,TGT,TCG,AGA,TTA,TTC,GCA,TCT,TTG,GCC,TTT,TCT,TGT,AAC,GTA,GCA,TTC |
| *OYE3*-13 | CTC,GAA,CAT,AGT,TTG,ACT,AAA,GAC,GAC,ATT,AAA,CAG,TAT,ATC,AAG,GAT,TAC,ATC,CAT,GCG |
| *OYE3*-14 | TTT,CTA,CAC,CAT,CGG,CGC,CAG,CCG,CGA,TAG,AAT,TCT,TAG,CCG,CAT,GGA,TGT,AAT,CCT,TGA |
| *OYE3*-15 | TGG,CGC,CGA,TGG,TGT,AGA,AAT,TCA,TAG,CGC,CAA,TGG,GTA,CTT,GTT,GAA,TCA,GTT,CTT,GGA |
| *OYE3*-16 | CGT,TCC,GCC,GTA,TTC,GTC,GGT,CCT,CTT,ATT,AGA,ATG,TGG,ATC,CAA,GAA,CTG,ATT,CAA,CAA |
| *OYE3*-17 | CCG,ACG,AAT,ACG,GCG,GAA,CGA,TCG,AAA,ACA,GGG,CCC,GCT,TTA,CAC,TGG,AGG,TTG,TCG,ATG |
| *OYE3*-18 | CTC,AAA,CCC,ACC,CGT,TCA,GGA,CCG,ATA,GTT,TCG,ATA,AGA,GCA,TCG,ACA,ACC,TCC,AGT,GTA |
| *OYE3*-19 | CCT,GAA,CGG,GTG,GGT,TTG,AGG,TTG,TCG,CCG,TAC,GGC,ACT,TTT,AAC,AGT,ATG,TCT,GGG,GGT |
| *OYE3*-20 | CCA,AAA,CAT,ACG,AAT,ATT,GAG,CGA,TAA,TAC,CTG,GTT,CAG,CAC,CCC,CAG,ACA,TAC,TGT,TAA |
| *OYE3*-21 | TCA,ATA,TTC,GTA,TGT,TTT,GGG,TGA,ATT,AGA,GAA,GAG,GGC,AAA,GGC,TGG,TAA,GCG,TTT,GGC |
| *OYE3*-22 | CGA,TGG,GTC,CGT,GAC,ACG,TGG,TTC,AAC,GAG,GTG,CAC,AAA,GGC,CAA,ACG,CTT,ACC,AGC,CTT |
| *OYE3*-23 | CAC,GTG,TCA,CGG,ACC,CAT,CGT,TGG,TGG,AGG,GCG,AAG,GAG,AAT,ATT,CCG,AGG,GTA,CTA,ACG |
| *OYE3*-24 | GCT,CTG,ATG,ATT,GGA,CCC,TTC,CAT,ATA,GAG,TAG,GCA,AAA,TCG,TTA,GTA,CCC,TCG,GAA,TAT |
| *OYE3*-25 | AAG,GGT,CCA,ATC,ATC,AGA,GCT,GGT,AAT,TAC,GCT,CTT,CAT,CCA,GAA,GTG,GTT,AGA,GAA,CAA |
| *OYE3*-26 | AGA,ATC,TAC,CAT,AGC,CTA,TCA,AGG,TTC,TGG,GAT,CCT,TTA,CTT,GTT,CTC,TAA,CCA,CTT,CTG |
| *OYE3*-27 | GAT,AGG,CTA,TGG,TAG,ATT,CTT,CAT,CTC,TAA,CCC,AGA,TTT,AGT,CTA,CCG,TTT,AGA,AGA,GGG |
| *OYE3*-28 | GGT,GTA,GAA,GGT,ACT,TCT,GTC,ATA,CTT,GTT,CAA,TGG,CAG,GCC,CTC,TTC,TAA,ACG,GTA,GAC |
| *OYE3*-29 | ACA,GAA,GTA,CCT,TCT,ACA,CCA,TGT,CCG,CGG,AAG,GTT,ATA,CCG,ACT,ACC,CAA,CAT,ATG,AAG |
| *OYE3*-30 | TCA,GTT,CTT,GTT,CCA,ACC,TAA,ATC,TAC,TGC,CTC,TTC,ATA,TGT,TGG,GTA,GTC,G AA,G, **GAG**,**CTC** |

Odd number: forward primer, Even number: reverse primer, Boldface: *Bam* HI and *Sac* I sites.
